# Defining and predicting service utilisation in young adulthood following childhood treatment of an eating disorder

**DOI:** 10.1192/bjo.2020.13

**Published:** 2020-04-06

**Authors:** Jessica McClelland, Mima Simic, Ulrike Schmidt, Antonia Koskina, Catherine Stewart

**Affiliations:** South London and Maudsley NHS Foundation Trust, UK; and Institute of Psychiatry, Psychology and Neuroscience, King's College London, UK

**Keywords:** Anorexia nervosa, bulimia nervosa, eating disorders NOS

## Abstract

**Background:**

Eating disorder services are often separated into child and adolescent eating disorder services (CAEDSs) and adult eating disorder services (AEDSs). Most patients in CAEDSs present with first-episode illness of short duration, which with appropriate treatment, have a good prognosis. However, some individuals receive further treatment as adults. Little is known about service utilisation in adulthood following childhood/adolescent treatment of an eating disorder.

**Aims:**

This study aims (a) to estimate the proportion of patients in a CAEDS who use mental health services as young adults, (b) to delineate service utilisation following treatment in CAEDSs and (c) to identify factors in CAEDSs that predict service utilisation in young adulthood.

**Method:**

A consecutive cohort of 322 patients (aged 13–17 years) seen in a CAEDS in the UK over a 5-year period were included in this audit. Data regarding their use of UK-wide adult mental health services as young adults (i.e. when aged 18–25) were extracted from local and national hospital records.

**Results:**

A total of 68.3% of CAEDS patients received no mental health treatment as young adults. Although 13% of people seen in a CAEDS had brief eating disorder treatment as young adults, 10% received longer/or more intensive eating disorder treatment. Overall, 10.8% transitioned directly to an AEDS and 7.6% were re-referred following discharge from CAEDS. In our sample, older age and increased use of CAEDSs predicted increased eating disorder treatment in young adulthood.

**Conclusions:**

Our results indicate that most people seen in CAEDSs do not receive further mental health treatment as young adults. Several features in CAEDSs distinguish mental health service utilisation in young adulthood, which were identified clinically and could be targeted during treatment.

Eating disorders are common and disabling mental disorders associated with high mortality rates.^1^ The peak age of eating disorder onset depends on diagnosis, typically being between 10 and 19 years of age^2^ and eating disorders often last between 5 and 8 years.^3^ The experience of an eating disorder during adolescence has been shown to predict poor psychiatric outcomes and high-risk behaviour in early adulthood.^4,5^ However, outcomes following skilled, specialist treatment for an eating disorder during childhood/adolescence are generally good (i.e. 75% of children/adolescents show clinically significant and lasting improvements^6–8^). Nevertheless, a considerable proportion of people continue to experience significant symptomology (such as low weight, psychopathology, bingeing, purging, depression) and key predictors of outcome have been identified (for example age at onset, duration of illness and weight status in anorexia nervosa^8–10^). Considering such research, many young people treated in specialist child and adolescent eating disorder services (CAEDSs) are likely to require further mental health treatment as adults. However, there is a lack of research regarding service utilisation in adulthood following treatment in CAEDSs. This may be because of difficulties arising from the distinct division of child and adolescent mental health services (CAMHS) and adult mental health services (AMHS^9,11^) that exists in many countries (such as UK, USA and Australia) or because many young people move away from home and are seen in adult services different to those attended in childhood.^12^

Given such difficulties, only three studies regarding the transition/pathways between CAMHS and AMHS in relation to eating disorders exist. Two of these are qualitative studies exploring clinician and patient views of transitioning from CAEDSs to AEDSs.^13,14^ There is only one existing study that quantitatively examines eating disorder services use from childhood to adulthood. In young adults referred to an AEDS, Arcelus et al^15^ found that approximately 28% had previously been seen in CAMHS and more than half of those individuals had received in-patient treatment for their eating disorder. Clearly, there is a paucity of research regarding AMHS use following treatment in CAEDS. An improved understanding of such service utilisation, including what features may predict increased treatment in AMHS, could inform improvements to service provisions in CAEDSs (for example personalised treatment targets), AEDS (such as consideration of optimal transitions) and possibly long-term outcomes (for example reduce extended service utilisation). The present study aims to (a) identify the proportion of people seen in a CAEDS who are later seen in AMHS, (b) to delineate service utilisation in adult services (such as no contact/brief/extended eating disorder treatment and/or other mental health treatment) and (c) to identify features in CAEDSs that are predictive of service utilisation in young adulthood.

## Method

This is a retrospective service-focused study therefore ethical approval was not required. The CAMHS Audit Committee from South London and Maudsley (SLaM) National Health Service (NHS) Foundation Trust approved the audit required for the project.

### Participants

Our audit identified children/adolescents treated at the Maudsley Centre for Child and Adolescent Eating Disorders (MCCAED) at SLaM NHS Foundation Trust, London, UK (covering a catchment area of >2 million local population plus some national, non-local referrals of individuals with complex/severe cases) over a 5-year period (2009–2014). In order to only include individuals from MCCAED who would be eligible (i.e. turn 18 years old) to use AMHS within the audit follow-up period (1 January 2013 until 31 July 2017), we staggered the inclusion criteria as follows: those seen in MCCAED who were aged ≥13 years in 2009, ≥14 years in 2010, ≥15 years in 2011, ≥16 years in 2012 and ≥17 years in 2013 (for the proportion assessed at each age, see supplementary material A available at https://doi.org/10.1192/bjo.2020.13). Therefore, the possible duration of time that included individuals who could have utilised AMHS ranged from 4 to 8 years (i.e. an 18-year-old seen in MCCAED in 2013 would be followed for 4 years, whereas an 18-year-old seen in MCCAED 2009 would be followed for 8 years). This meant that AMHS use considered in this study was in relation to a young adult sample (i.e. aged 18–25 years).

### Procedures

Eligible individuals and their relevant data were identified and extracted from three sources – an internally held database within MCCAED, the Clinical Record Interactive Search (CRIS) system (which provides anonymised information extracted from SLaM NHS Foundation Trust electronic clinical records) and the Hospital Episode Statistics (HES) system (which enables acquisition of data from all NHS hospitals in England pertaining to countrywide mental health related admissions, out-patient appointments and accident and emergency (A&E) attendance). HES data allowed for the follow-up of both national/non-local patients and of local patients who may have left the area (for example to attend university). Data from HES were non-specific to the type of mental health treatment/service, therefore are referred to as ‘unspecified mental health treatment’. From these three sources, data pertaining to demographic, service utilisation and clinical information were extracted. Data were initially extracted from the MCCAED database, which has all identifiable information removed and uses unique non-identifiable numbers. SLaM identifications that related to the non-identifiable numbers were then provided to the CRIS team, who extracted and matched the additional data required from CRIS and HES. The CRIS team then applied a new non-identifiable number to each data-set, to ensure data could not be retrospectively traced.

### Measures

In order to compute categories of AMHS use and predictors of these, data regarding MCCAED and AMHS use were extracted as well as several measures at MCCAED assessment and discharge.

#### Treatment characteristics (type, duration, attendance)

Details of treatment contracts provided information relating to the type (for example out-patient, day-patient, in-patient), duration (for example start and end date) and attendance (for example number of psychological sessions attended) of treatment in the SLaM MCCAEDS, AEDS, non-eating disorder adult SLaM services and non-local unspecified AMHS. Countrywide presentations to A&E were also extracted as well as information regarding eating disorder and other diagnoses during both childhood and young adulthood (for example A&E presentation and diagnoses recorded pre and post 18 years of age).

#### Percentage median body mass index

Percentage median body mass index (%mBMI) was calculated at MCCAED assessment and discharge and relates to the BMI of a child relative to the average BMI of comparable children, expressed as a percentage.

#### Eating Disorder Examination Questionnaires

Version 6 of the Eating Disorder Examination Questionnaires (EDE-Q^16^) was completed at MCCAED assessment and discharge. Questions relate to eating disorder symptoms over the past 28 days. Four subscale scores are calculated as well as a global score. High scores indicate worse eating disorder psychopathology, with a proposed clinical cut-off global score of ≥2.8.

#### Mood and Feelings Questionnaire

The short version of the Mood and Feelings Questionnaire (MFQ^17^) was completed at MCCAED assessment. Questions relate to symptoms of depression and anxiety over the past 2 weeks and are rated ‘not true’, ‘sometimes true’ or ‘true’. Scores range from 0 to 26, with a score of ≥12 indicating the presence of depressive symptoms.

#### Screen for Child Anxiety Related Disorders

The child-rated version of Screen for Child Anxiety Related Disorders (SCARED^18^) was completed at MCCAED assessment. It includes 41 items relating to symptoms of anxiety over the past 3 months rated ‘not/hardly ever true’, ‘somewhat/sometimes true’ or ‘very/very often true’. Subscales relating to different anxiety disorders are generated and a total score of ≥25 indicates the presence of an anxiety disorder.

#### Obsessive-Compulsive Inventory

The Obsessive-Compulsive Inventory (OCI^19^) was completed at MCCAED assessment, which includes 42 questions relating to symptoms of obsessive–compulsive disorder (OCD) over the past month. Each item is rated from 0 ‘not at all’ to 4 ‘extremely’, used to calculate seven subscale scores as well as an overall mean distress score. A total score of ≥42 suggests the presence of OCD.

#### Children's Global Assessment Scale

The Children's Global Assessment Scale (CGAS^20^) was completed at MCCAED assessment and discharge. The CGAS is a rating of overall functioning provided by the primary clinician in relation to a child's psychological and social functioning. Scores range from 0 (‘extremely impaired’) to 100 (‘doing very well’).

### Outcomes

In order to delineate and predict adult service utilisation, data were extracted and outcome variables created, as detailed here.

#### Proportion seen in adult mental health services

Data regarding duration and type of treatment in the SLaM AEDS; non-eating disorder adult SLaM services and non-local unspecified AMHS were used to calculate the proportions of young adults who were seen in AMHS overall and the SLaM AEDS specifically.

#### Delineation of adult service utilisation

Categories of adult service utilisation were defined by lead clinicians from the MCCAED and AEDSs (authors C.S., U.S. and M.S.), including definitions for no, low, medium and high use of AEDSs (see supplementary material B). These were based on duration of in-patient eating disorder treatment, duration and/or number of psychological treatment sessions attended during day-patient/out-patient eating disorder treatment, as well as attendance at A&E as a young adult. A small proportion of individuals did not fall into these categories as they received only non-eating disorder treatment as young adults in SLaM or unspecified treatment in AMHS other than SLaM, these were categorised separately and accordingly.

#### Prediction of adult service utilisation

Data pertaining to age at referral to MCCAED; use of MCCAED (for example duration and type of eating disorder treatment); eating disorder severity (%mBMI, EDE-Q), comorbidity (MFQ, SCARES, OCI) and global functioning (CGAS) at initial MCCAED assessment and attendance at A&E as a child/adolescent were used as predictors of adult service utilisation. Given a range of the available data were relevant to factors relating to MCCAED use (in-patient, day-patient, out-patient), eating disorder severity (%mBMI, EDE-Q scores) and comorbidity (mood, anxiety, obsessive/compulsive symptoms), composite scores were created for these predictor variables (see supplementary material C).

Some data were unable to be used as predictor variables because of inconsistencies in record keeping (such as risk of suicide/self-harm) and/or uneven distribution of data (for example higher proportion of people with a diagnosis of anorexia nervosa).

### Statistical analyses

Statistical analyses followed the three study aims (a) descriptive analyses to present the demographic, clinical and service utilisation characteristics of the participants, specifically the proportion of young adults later seen in AMHS; (b) Kruskal–Wallis and χ^2^-tests to delineate young adult service utilisation and (c) generalised linear model for ordinal outcomes to predict service utilisation in young adulthood based on features at MCCAED assessment. The generalised linear model relates to three broad outcome groups of AEDS use in young adulthood, i.e. no contact, brief (low and medium AEDS pathways combined) and extended treatment (high use) in AEDS. Key assumptions for all analyses (for example linearity, normality, multicollinearity, independence of errors, autocorrelation and homoscedasticity) were explored and addressed where necessary.

## Results

### Participants

A total of 322 people who were treated by MCCAED were eligible for this audit (i.e. aged between 13 and 17 years at MCCAED assessment and therefore able to use AMHS as young adults during the follow-up period). This sample is approximately 65% of all young people treated and 50% of all referrals made to MCCAED during the specified 5-year period. The majority were White British (83%), 3% were of Asian origin and the remaining 14% had other ethnic backgrounds. The majority (82.7%) were referred from the local catchment area. These participants did not differ from the individuals who were national/non-local referrals at assessment in age, or self-reported symptoms of eating disorder, mood or anxiety (all *P* > 0.05). Those diagnosed with a restrictive eating disorder did not differ in %mBMI at baseline assessment. Those who were national/non-local referrals were less likely to be diagnosed with a binge/purge type eating disorder.

### Proportion seen in adult mental health services

**Fig. 1:**
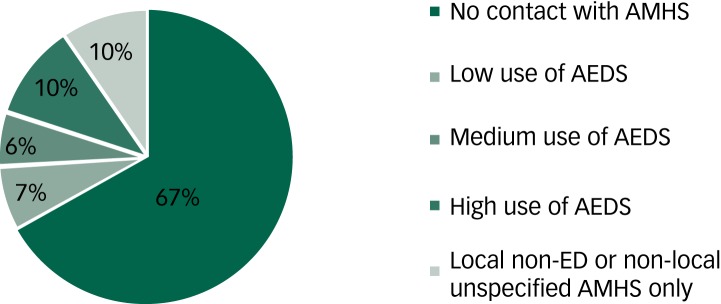
Proportion of people seen in adult services following child/adolescent treatment of an eating disorder.

AMHS, adult mental health service; AEDS, adult eating disorder service; ED, eating disorder.

Figure 1 demonstrates the proportion of people seen in MCCAED who were later seen in AMHS as young adults. Two-thirds of this sample had no contact with AMHS, however, one-third received further mental health treatment as young adults. In 13% of this sample, brief eating disorder treatment was received (such as low/medium use of the AEDS involving <20 out-patient treatment sessions) whereas 10% had extended eating disorder treatment (for example high use of AEDSs involving in-patient treatment and/or over 20 out-patient treatment sessions). The remaining 10% of these young adults were seen for local (SLaM) non-eating disorder mental health treatment only (2.2%) or were seen by non-local NHS AMHS (i.e. not SLaM, 7.4%) for unspecified mental health treatment.

Approximately 20% of the young people seen in MCCAED received in-patient treatment. Of the young adults later seen in the AEDS, 32% had received in-patient treatment as children/adolescents. Additionally, 10.8% and 7.6% of those seen in MCCAEDS were referred directly to AEDS or re-referred following discharge by MCCAEDS back to their general practitioner, respectively. In terms of the proportion of young adults who were later seen in AEDS, 47% were referred directly from MCCAEDS, while 33% were initially discharged by MCCAEDS back to their GP and then re-referred to AEDS.

### Delineation of adult service utilisation

As indicated in Tables 1 and 2, Kruskal–Wallis and χ^2-^tests indicated that there were distinctions between adult service utilisation during young adulthood based on several features in MCCAED and AEDS, as described below.

**Table 1:** Factors in childhood/adolescence that distinguished young adult service utilisation (median and ranges reported unless indicated)

	No contact with AMHS	Low use of AEDSs	Medium use of AEDSs	High use of AEDSs	Local non-eating disorder AMHS	Non-local unspecified AMHS	*P*
Age, years: median (IQR)	16 (15–17)	16 (15–17)	16 (15–17)	17 (15–17)	16 (15–17)	16 (16–17)	**0.03**
Disorder, %							**<0.01**
Anorexia nervosa	35	32	42	68	0	46	
Bulimia nervosa	11	32	5	12	43	0	
Eating disorder not otherwise specified	37	23	5	18	57	46	
Unspecified feeding or eating disorder	17	14	47	3	14	8	
Comorbid diagnoses, median (range)	1 (0–4)	1 (0–5)	1 (0–5)	1 (0–7)	2 (0–7)	1 (1–4)	0.215
Untreated eating disorder, months: median (range)	12 (3–84)	24 (5–48)	7 (2–60)	10 (3–65)	11 (3–78)	21 (2–72)	**0.01**
%mBMI at assessment, median (range)	84 (54–133)	85 (70–126)	82 (70–100)	79 (55–98)	98 (95–119)	82 (65–98)	**<0.01**
EDE-Q at assessment, median (range)	3.7 (0–5.9)	3.8 (0.9–5.6)	3.8 (0–5.4)	3.9 (0.3–5.7)	4.7 (1–5.2)	4.2 (0.3–5.7)	0.24
SCARED at assessment, median (range)	28 (0–77)	26 (2–58)	33 (0–49)	38 (9–67)	40 (15–58)	38 (6–68)	0.06
CGAS at assessment, median (range)	45 (21–90)	45 (31–65)	40 (35–55)	41 (27–65)	55 (40–65)	41 (22–67)	0.15
In-patient, months: median (range)	1.8 (0–15)	4.2 (1–8)	2.3 (1–10)	3.3 (1–28)	0.8 (0–1)	3.1 (1–17)	0.07
All treatment, months: median (range)	8 (1–50.1)	10 (2–24)	10 (2–37)	10 (1–64.5)	6 (1–21)	11 (2–21)	0.37
A&E attendance, median (range)	1 (0–32)	1 (0–12)	1 (0–7)	2 (0–19)	2 (0–7)	1.5 (0–11)	0.62
%mBMI at discharge, median (range)	93 (66–163)	94 (74–115)	91 (73–125)	82 (64–120)	104 (87–118)	86 (65–104)	**<0.01**
EDE-Q at discharge, median (range)	1.0 (0–6)	1.4 (0.3–4.5)	2.6 (0.6–4.1)	3.1 (0.8–5.6)	3.1 (3.1–3.1)	3.9 (2.9–5.6)	**<0.01**
CGAS at discharge, median (range)	70 (34–98)	62 (31–90)	60 (45–70)	56 (34–99)	55 (46–70)	61 (30–90)	**<0.01**

Results in bold are significant. AMHS, adult mental health service; AEDS; adult eating disorder service; %mBMI, percentage medium body mass index; EDE-Q, Eating Disorder Examination Questionnaire; SCARED, Screen for Child Anxiety Related Disorders; CGAS, Children's Global Assessment Scale; A&E, accident and emergency.

**Table 2:** Factors in young adulthood that distinguished young adult service utilisation

	No contact with AMHS	Low use of AEDSs	Medium use of AEDSs	High use of AEDSs	Local non-eating disorder AMHS	Non-local unspecified AMHS	*P*
Disorder, %^a^							**0.01**
Anorexia nervosa	35	45	47	79	14	17	
Bulimia nervosa	25	14	10	8	14	4	
Eating disorder not otherwise specified	5	18	10	12	14	4	
Unspecified feeding or eating disorder	35	23	32	1	56	75	
Comorbid diagnoses,^a^ median (range)	1 (1–3)	1 (1–5)	1 (1–6)	2 (1–5)	3 (2–5)	1 (1–2)	**<0.01**
In-patient treatment, months: median (range)	–	–	2.0 (1–3)	6.8 (2–15)	–	0.7 (0–19)	**0.02**
SLaM day care/out-patient treatment, months: median (range)	–	2.9 (0–9)	7.6 (4–21)	16.6 (0–58)	–	8.6 (0–49)	**<0.01**
SLaM out-patient appointments attended,^a^ median (range)	3 (1–4)	1 (1–6)	15 (1–20)	34 (2–116)	14 (6–23)	1 (1–14)	**0.02**
A&E attendance,^a^ median (range)	2 (1–19)	2 (1–4)	1 (1–6)	2.5 (1–15)	4 (2–8)	4 (1–32)	**<0.01**

Results in bold are significant. AMHS, adult mental health service; AEDS; adult eating disorder service; SLaM, South London and Maudsley; A&E, accident and emergency.

a. Documented after an individual turned 18 years of age.

#### Factors in MCCAED that distinguished adult service utilisation

Age, duration of untreated eating disorder, clinical presentation at MCCAED assessment (i.e. eating disorder diagnosis, %mBMI and a significant trend for SCARED scores), duration of in-patient treatment (trend level only) and clinical features at MCCAED discharge (such as age, reason for discharge, %mBMI, EDE-Q, CGAS) were significantly different according to adult service utilisation. Young adults in the ‘high use’ of AEDS category were more likely to have a diagnosis of anorexia nervosa, were older at assessment and had a lower weight at MCCAED assessment and discharge, had longer CAMHS in-patient treatment, worse assessment SCARED scores and worse EDE-Q and CGAS scores at discharge from MCCAED. Also, a significantly larger proportion (χ^2^ (20) = 108.68, *P* < 0.001) of young adults in the ‘high use’ of AEDS were discharged from MCCAED directly to AEDSs, i.e. 63% *v.* <50% in other groups. Young adults in the ‘low use’ of AEDSs had longer duration of untreated eating disorder compared with other adult service utilisation groups. The number of comorbid diagnoses; eating disorder, other psychopathology (i.e. OCI, MFQ) and overall functioning (CGAS) at assessment; duration of all treatment (i.e. in-patient, day and out-patient) and A&E attendance during childhood/adolescence were not significantly different across the different adult service utilisation groups.

#### Factors in AEDS that distinguished adult service utilisation

As expected and consistent with our predefined adult service utilisation groups, these significantly differed from one another according to use of AEDSs (for example length of in-patient, day-patient, out-patient treatment; number of psychological out-patient sessions attended), presentation to A&E and diagnoses (such as eating disorder; comorbidity; see Table 2). As well as increased uptake of eating disorder treatment, young adults in the ‘high use’ of AEDSs category also presented to A&E more frequently, were more likely to have anorexia nervosa and comorbid diagnoses. Increased number of comorbid diagnoses and presentation to A&E were also characteristic of the local non-eating disorder AMHS category, as well as increased presentation to A&E in the non-local unspecified AMHS category.

### Factors predictive of adult service utilisation

A generalised linear model for ordinal outcomes significantly predicted extended treatment in AEDSs (χ^2^ (6) = 35.91, *P* < 0.001). Table 3 reports regression statistics for each of the predictor variables included in the model.

**Table 3:** Generalised linear model for the effect of factors in childhood/adolescence in predicting young adult service utilisation

	Odds ratio	95% CI	*P*
Age at referral to MCCAED	1.69	1.26–2.33	**0.001**
Service use in MCCAED	1.43	1.22–1.68	**<0.001**
Eatingdisorder severity at MCCAED assessment	1.21	0.98–1.51	0.07
Comorbidity at MCCAED assessment	1.03	0.87–1.24	0.70
CGAS at MCCAED assessment	0.99	0.96–1.03	0.77
A&E attendance as a child/adolescent	0.96	0.86–1.05	0.46

Results in bold are significant. MCCAED, Maudsley Centre for Child and Adolescent Eating Disorders; CGAS, Children's Global Assessment Scale; CAEDS, child and adolescent eating disorder service; A&E, accident and emergency.

As indicated in Table 3, if the age a child/adolescent was referred to MCCAED increased by 1 (year), their ordered log-odds of receiving extended treatment in AEDS as a young adult would have a multiplicative increase of 1.69, while the other variables in the model are held constant. Similarly, if a child/adolescent were to increase their use of MCCAED services by 1 point (in relation to composite scores defined in supplementary material C), their ordered log-odds of receiving extended treatment as a young adult in AEDSs would have a multiplicative increase of 1.43, while the other variables in the model are held constant. The effect of increased eating disorder severity on receiving extended treatment in AEDSs as a young adult approached levels of significance, however, comorbidity and global functioning (CGAS) at MCCAED assessment, as well as use of A&E services during childhood/adolescence, did not predict increased use of AEDS.

## Discussion

This is the first study to examine service utilisation in AMHS following treatment in a specialist CAEDS. Our findings are discussed in relation to our three aims and relevant literature.

In relation to our first aim, we found that although the majority of MCCAED patients do not receive treatment from AMHSs, one-third required further mental health treatment as young adults and 23% required treatment in an AEDS. This is similar to findings reported in another AEDS regarding previous eating disorder treatment in CAMHS (28%^15^). Overall, a small proportion transitioned from MCCAED to AEDSs directly or were re-referred following discharge from MCCAEDS (10.8% and 7.6%, respectively). However, during the period audited there was not an automatic transfer between MCCAED and AEDSs, therefore this number is skewed by a proportion of young adults who would have been discharged to their general practitioner with a request for them to refer to the AEDS. Therefore, the current data overestimates the number of young people considered ‘recovered/remitted’ who then ‘relapsed’ and underestimates the number who were in transition between child and adult services. Nonetheless, our findings are more optimistic than the notion that up to 50% of young people have residual morbidity (in eating disorders and other areas such as depression and anxiety) after their eating disorder has remitted.^21–23^ In relation to in-patient treatment, almost one-third of the young adults who were seen in AEDSs had received in-patient treatment as children/adolescents, which was a greater proportion than the overall CAEDS in-patient admission rate. However, this is a lesser proportion (58%) than previously reported by Arcelus et al.^15^

In relation to our second aim, several factors in MCCAEDS distinguished adult service utilisation in young adulthood. For example, no/brief use of AEDSs was associated with the longest duration of untreated eating disorder. This is likely to reflect the higher proportion of young adults who had non-anorexia nervosa or milder eating disorders (bulimia nervosa, eating disorder not otherwise specified or unspecified feeding or eating disorder) as children/adolescents, as these groups typically present to CAEDSs later than those with anorexia nervosa. Extended use of AEDSs in young adulthood was associated with older age at MCCAED assessment and worse symptomatology at presentation/discharge from MCCAED. These findings may relate to our sampling methodology that captured an older, more transitional group of children/adolescents seen in MCCAED. Additionally, they may reflect that children/adolescents who attend CAEDSs later (i.e. at an older age) or individuals with more severe illness are those that go on to receive extended treatment in AEDSs during young adulthood.

Finally, several findings relating to adult service utilisation categories may reflect more complex presentations in these groups. For example, young adults that had extended treatment in AEDSs were more likely to have anorexia nervosa, comorbid diagnoses and attend A&E more frequently. Additionally, comorbid diagnoses and presentation to A&E, as well as just presentation to A&E were characteristic of young adults in the local non-eating disorder AMHS group (i.e. involving comorbid treatment only) and the non-local unspecified AMHS group (i.e. likely to include national patients with severe and complex cases). These features are possibly indicative of physical complications associated with low weight, emergency and risk-related service use. Such factors are associated with longer-term mental health conditions such as personality disorders,^24,25^ which when they coexist with eating disorders, are associated with poorer outcomes.^26^ Overall, these findings highlight the input from a range of AMHS during young adulthood following treatment for an eating disorder in childhood/adolescence.

In relation to our third aim, we found that two features in MCCAED predicted extended treatment in AEDSs for young adults. First, older age at presentation to MCCAED, although this may be explained by our sampling method, which captured an older subset of total referrals to MCCAED. However, if children/adolescents are older at presentation to CAEDSs, they may have less time to receive adequate treatment for their eating disorder and/or be less likely to receive a full course of a family-based treatment approach before needing to be transferred to AEDSs. These may have an impact on speed and rates of remission for these individuals. Related to this, the organisational structure of the UK National Health Service requires young people to be transferred from CAMHS to AMHS at the age of 18 years, irrespective of stage of illness/recovery. If this transition happens at a crucial stage of illness, as indicated by our reported differences in clinical characteristics at discharge from MCCAED across adult service use categories, this could have detrimental effects on treatment outcomes for young adults in AEDSs. The second factor found to predict increased use of AEDSs in young adults was increased use of MCCAED (i.e. length and number of in-patient, day-patient and/or out-patient episodes). This resonates with findings by Hergenroeder et al^27^ indicating that repeated admissions for eating disorder treatment during adolescence are associated with poor recovery. These findings highlight clinical identification in CAEDS of the likelihood for ongoing treatment into young adulthood and supports the notion that quick resolution of eating disorders in childhood/adolescence is optimal. Interestingly, eating disorder severity in CAEDSs may also predict extended treatment in AEDSs in young adults, which is in line with the predictive nature of increased use of MCCAED on AEDS use. Comorbidity, overall functioning and presentation to emergency services during childhood/adolescence did not. This may be explained by insufficient sample size, our methodology (such as sampling, analyses) and/or an inadequate ability to capture clinical features that predict persistence/chronicity. One possible solution to this is to also include outcomes at discharge from CAEDSs as predictors of young adult service utilisation. Overall and as mentioned above, children/adolescents attending CAEDSs at an older age or those that have severe eating disorder requiring more treatment in CAEDSs, are those that are likely to require extended treatment in AEDSs as young adults.

### Limitations

Our study describes AMHS utilisation in young adults who had previous eating disorder treatment in a CAEDS. It is important to consider the distinction between service utilisation and clinical need for further treatment in young adulthood when interpreting these findings. As mentioned, the sampling method of this retrospective audit study meant that this was an older, more transitional subsample of the children/adolescents typically seen in CAEDSs. This limits the reliability of our finding regarding the predictive nature of age on use of AEDSs as well as the generalisability of our results, for example to people presenting to CAEDSs who are under 13 years of age. Additionally, although the national referrals included in this sample were not found to differ from the local group, they are likely to have been individuals with more severe, treatment-resistant illness with unique prognostic factors. Their inclusion limits generalisability to other settings. The conclusions that can be drawn over the proportion of people considered recovered or remitted and discharged to the general practitioner who later relapsed as young adults are limited by the lack of transition procedures in place during the audit period. Unfortunately, we were not able to extract data regarding reasons for discharge, duration between discharge from MCCAED and re-referral to AEDSs, or the age at which young adults were re-referred. This information would have been useful in understanding the proportion and characteristics of young people who did not respond to treatment in MCCAED, versus those who did and then relapsed. Moreover, individuals were followed for up to 8 years, therefore we are not able to evaluate response to treatment and/or relapse rates beyond 25 years of age. Related to this, the anonymised nature of this audit data meant that we were unable to follow-up individual patients to gather additional information, for example circumstances around re-referral from general practitioners to AEDSs.

Our retrospective audit methodology limited the sample size that we could extract and evaluate, which decreased statistical power and had an impact on analyses. Inconsistencies in record keeping (for example clinical risk) and uneven distribution of data (for example larger proportion of children/adolescents seen in CAEDSs with anorexia nervosa compared with other eating disorders) meant that data pertaining to key clinical features were unattainable and/or unable to be included in analyses. The estimate of duration of untreated eating disorder is a clinical judgment based on brief subjective reports from families during assessment and is therefore limited. Additionally, limitations in data extraction methodology (such as specificity of HES data regarding non-local AMHS use) meant that we were unable to specify what type of mental health treatment a significant proportion of children/adolescents seen in CAEDSs went on to receive as young adults. This restricted analyses accordingly (for example limiting the predictive model to AEDSs only, rather than all AMHS use). Despite our inclusion of countrywide AMHS use, we may have missed those seen in CAEDSs who were discharged back to primary care and may have been managed in other less intensive/specialist mental-health related support services as young adults (such as student services, self-help groups^9,28^). Finally, unlike others^15^ our study did not include clinical measures at presentation to AEDSs, which could have been informative.

### Implications

Despite this study's limitations, there are several important implications of our findings. First, improved transitions for older adolescents presenting to CAEDS and those with more severe forms of illness should be developed. Second, features in CAEDS that indicate increased use of AEDSs in young adulthood (for example increased levels of anxiety at presentation) could be better identified and incorporated as personalised treatment targets while in CAEDSs. Third, factors that contribute to repeated adolescent in-patient admissions as well as extended treatment/poor response during out-patient treatment in CAEDSs, could be better understood. It will be equally useful for treatment in AEDSs to be informed by the often-numerous in-patient admissions during childhood/adolescence. Additionally, many young adults in AEDSs who have been transferred from CAEDSs may have done so at a crucial stage of their illness whereas those that have been re-referred to specialist eating disorder services via their general practitioner may have been discharged from CAEDSs prematurely. These factors have important implications for improving transitions and engagement of young adults within AEDSs. As others have argued,^9,11^ the importance of improving the transition between CAEDSs and AEDSs is a key area for future service developments (such as staff working across services, trans-age services, illness-based transition flexibility). Along with the ideas for future research already mentioned, prospective studies that follow young adults’ journeys after treatment in CAEDSs are needed. These should include detailed information regarding types of mental health service, treatment and support accessed as young adults and beyond.

In conclusion, this study has highlighted that most people seen in a CAEDS do not go on to receive treatment in AMHS as young adults. However, a significant proportion of children/adolescents who receive treatment for an eating disorder receive further mental health treatment as young adults, either for enduring illness or because of relapse. Several features distinguish those who receive extended treatment in AEDSs as young adults, including older age, lower weight, worse psychopathology and overall functioning at presentation to and/or discharge from CAEDSs. This is the first study to report the predictive nature of older age at presentation to CAEDSs and increased use of CAEDSs on extended treatment in AEDSs during young adulthood. These findings imply that children/adolescent who present to CAEDSs when they are older or those that have more severe forms of eating disorders are those who are likely to receive increased input from AEDSs as young adults. This reflects the notion that an adequate course of specialist treatment is required for all eating disorders as well as the continuity of more severe, complex eating disorders. This study demonstrated the clinical identification of ongoing need and the importance of considering such factors during treatment in CAEDSs. Longitudinal, prospective studies are needed to explore other predictive factors of AMHS use following childhood treatment of an eating disorder and the impact of targeting these during treatment.

## Data Availability

As this study involved a retrospective audit, the authors have full and ongoing access to the data.

## Supplementary material

For supplementary material accompanying this paper visit http://dx.doi.org/10.1192/bjo.2020.13. click here to view supplementary material
