# Supplementary material for: Defining and predicting service utilisation in young adulthood following childhood treatment of an eating disorder
Source: BJPsych Open. 2020 Apr 6;6(3):e37. doi: 10.1192/bjo.2020.13 (PMC7176893; doi:10.1192/bjo.2020.13)
Supplement: Supplementary file 1 [file S2056472420000137sup001.docx]

**Supplementary material A – Proportion of sample based on age at initial assessment.**

| **Age at CAEDS assessment (years)** | 13 | 14 | 15 | 16 | 17 |
| --- | --- | --- | --- | --- | --- |
| **Number in sample** | 14 | 39 | 66 | 89 | 115 |
| **Proportion of sample (%)** | 4 | 12 | 21 | 28 | 35 |

**Supplementary material B – Factors defining young adult service utilisation.**

|  | **No contact with adult services** | **Low use of AEDS** | **Medium use of AEDS** | **High use AEDS** | **Local non-ED AMHS** | **Non-local unspecified AMHS** |
| --- | --- | --- | --- | --- | --- | --- |
| Young adult inpatient ED treatment | - | - | - | > 0 months  **and/or** | - | Non-SLaM only, any duration |
| Young adult day-patient ED treatment | - | - | 3 – 6 months  **and/or** | > 6 months  **and/or** | - | Non-SLaM only, any duration |
| Young adult outpatient ED treatment | < 5 sessions | < 3 months  **or** < 10 sessions | 3 – 6 months  **or** 10-20 sessions | > 6 months  **or** > 20 sessions  **and/or** | - | Non-SLaM only, any duration |
| Young adult non-ED mental health treatment | Any value | Any value | Any value | Any value | Any value | Non-SLaM only, any duration |
| Attendance at A&E as a young adult | 0 | < 5 | < 10 | Any value | Any value | Any value |

AEDS: adult eating disorder service; AMHS: adult mental health service; ED: eating disorder; SLaM: South London and Maudsley NHS Foundation Trust; A&E: accident and emergency.

**Supplementary material C – Definitions of composite predictive factors.**

| **Score assigned** | **0** | **1** | **2** | **3** | **4** |
| --- | --- | --- | --- | --- | --- |
| Service use in MCCAED: | (total of four, range 0-16): | | | | |
| Inpatient stay: | 0 | < 1 month | 1-3 months | 4-6 months | > 6 months |
| Day-patient contracts: | 0 | 0 | 1 | 2 | > 2 |
| Outpatient contracts: | 0 | 1 | 2 | 3 | > 3 |
| Duration treatment: | 0 | 0-6.0 months | 6.1-12.0 months | 12.1 – 24.0 months | > 24 months |
| ED severity at MCCAED assessment: | (total of two, range 0-8): | | | | |
| EDE-Q: | < 1 | 1 – 1.99 | 2.0 – 2.99 | 3.0 – 4 | > 4 |
| %mBMI: | > 95 | 86 - 95 | 75 - 85 | < 75 |  |
| Comorbidity at MCCAED assessment: | (total of x, range 0-9): | | | | |
| MFQ: | 0 | 1-13 | 14-27 | > 27 |  |
| SCARED: | 0 | 1-11 | 12-24 | ≥ 25 |  |
| OCI: | <11 | 12-23 | 24-37 | ≥ 38 |  |

MCCAED: Maudsley Centre for Child and Adolescent Eating Disorder; ED: eating disorder; EDE-Q: Eating Disorder Examination Questionnaire; %mBMI: percentage medium BMI; MFQ: mood and feeling questionnaire; SCARED: Screen for Child Anxiety Related Disorders; OCI: Obsessive-Compulsive Inventory;
